# Exploring the association of calbindin –D28K in renal dialysis with oral health: a comprehensive review

**DOI:** 10.3389/froh.2025.1523024

**Published:** 2025-04-25

**Authors:** Mohamed Jaber, Mohamed Saleh Hamad Ingafou, Nireeksha, Alexander Maniangat Luke

**Affiliations:** ^1^ Department of Clinical Sciences, College of Dentistry, Ajman University, Ajman, United Arab Emirates; ^2^ Centre for Medical and Bio-Allied Health Sciences Research (CMBAHSR), Ajman University, Ajman, United Arab Emirates; ^3^ Department of Conservative Dentistry and Endodontics, AB Shetty Memorial Institute of Dental Sciences, Nitte (deemed to be) University, Deralakatte, India

**Keywords:** calbindin-D28k, renal dialysis, oral health, chronic kidney disease, REB

## Abstract

**Background:**

The kidney, brain, and endocrine glands all express calbindin-D28K, a calcium-binding protein that is essential for maintaining calcium homeostasis. Calcium metabolism is disturbed in chronic kidney disease (CKD), which may have an impact on dental and bone health. Patients on dialysis frequently have changed salivary composition, which raises their risk of dental problems such tooth decay and periodontal disease. Although there is no direct study on the relationship between Calbindin-D28K and dental health in dialysis patients, its function in calcium control raises the possibility of a connection that merits more investigation.

**Objective:**

To address the impact of Calbindin-D28K levels in chronic kidney disease on oral health.

**Design:**

A comprehensive electronic search was conducted using databases like PubMed, ResearchGate, SCOPUS, and others, to find relevant articles published before May 2024. The search terms included Calbindin-D28K, renal dialysis, dental and oral health, Vitamin D, calcium, end-stage renal disease, and related topics. The review examined studies from 1975 to 2024, focusing on the relationship between dental health and renal dialysis and factors affecting Calbindin-D28K levels.

**Results:**

A total of 48 articles were retrieved through electronic database. After evaluating the title, abstract, and full text of these articles, only 7 were selected for the present review. Final consideration: Based on the data available on selected studies, they point to a possible association between these people's higher frequency of periodontal disease and tooth caries and lower levels of Calbindin-D28K.It is imperative to recognize the reciprocal impact of systemic illnesses on oral health. In addition to being important for maintaining calcium homeostasis, calbindin-D28K may also be a biomarker for renal damage and have an impact on dental health. Its relevance for both diagnosis and treatment of chronic kidney disease and associated diseases is highlighted by its involvement in renal function and neuroprotection.

## 1 Introduction

Chronic kidney disease is a significant social and health problem that affects over 70,000 million people globally (1). As of right now, 8,724 out of one lakh people globally suffer with chronic kidney disease, which is ten times more common than cancer cases and more than 80% more advanced than diabetes (2). It is expected that by 2040, chronic kidney disease will rank as the fifth leading cause of death globally (1). Chronic kidney disease is mostly caused by diabetes, with glomerular diseases and hypertension.However, after a critical mass of nephron has been lost, kidney disease usually advances to more advanced stages, which are indicated by heightened morbidity and mortality, regardless of the underlying nephropathy The impact of chronic kidney disease complications and significant comorbidities, as well as the management of the disease, contribute to the burden of end-stage kidney disease (3).

An individual's general quality and well-being are influenced by their oral health. Tooth cavities, periodontitis, and other systemic disorders can be brought on by poor oral hygiene (4). Individuals with chronic kidney disease frequently have changes in their oral cavities, such as periodontitis and other signs of oral health problems. These changes can lead to increased rates of morbidity and death (5). Chronic kidney disease is characterized by progressive kidney damage that shows up as abnormalities in the structure and function of the kidneys that last for three months or more (6). The final stage of chronic kidney disease, known as end-stage renal disease, is divided into five phases based on albuminuria and glomerular filtration impairment. End-stage renal disease management requires renal replacement therapy, such as dialysis.

Patients receiving renal dialysis may be able to predict their health outcomes by their oral health. There is a higher risk of periodontal disease associated with poor dental hygiene (7). Notably, more severe oral diseases, caries, dry mouth, periodontitis, poor oral hygiene, and rising fatalities with changes in salivary content and pH, decreased salivary flow, increased dental calculus arrangement, increased salivary buffering capacity, urea, potassium, and phosphate levels, and a fall in calcium have all been reported in Chronic kidney disease patients with ESRD that are comparable to the general population (8). Calcium reabsorption is decreased in Chronic kidney disease, which also affects vitamin D and mineral metabolism. chronic kidney disease or its therapy can cause a number of changes in the oral cavity, even though there are no specific symptoms of the disease (9).

It has been demonstrated that chronic kidney disease damages teeth and ultimately results in their loss (10, 11); it also affects the oral mucosa (12), bone (13); periodontium (12, 14); salivary gland dysfunction (15), tongue (16), mouth cavity (17), and temporomandibular joint (37).Multiple research studies have found that dialysis patients have a higher rate of oral pathology (18) when they are experiencing one or more oral symptoms including xerostomia (19), dysgeusia (20), uremic scent (21), tongue coating (22), mucosal inflammation (23), mucosal petechia/ecchymosis, oral ulceration, or enamel hypoplasia (19, 24). Dry mouth can cause cavities and gingival inflammation as well as stuttering, denture retention, mastication, dysphagia, mouth ulcer, ageusia, and infections (12).

## 2 Materials and methods

Using various online databases including PubMed, Researchgate, SCOPUS, Elsevier, Web of Science, Google, ScienceDirect, and Google Scholar, a comprehensive electronic search was conducted before May 2024 to screen relevant articles for the purpose of the study. The search terms included “Calbindin –D28K” “renal dialysis” “oral health” “dental health” “Vitamin D” “calcium binding protein” “calcium” “End-stage renal disease” “calcium regulation” “periodontal health” and “salivary gland health”. The data from studies published between years 1975 and 2024 were assessed, examined, and the findings were collated in the current narrative review. A total of 48 articles were retrieved through electronic database. After evaluating the title, abstract, and full text of these articles, only 7 were selected for the present review. This study gathered and reviewed the relationship between dental health and renal dialysis, as well as the many factors that affect Calbindin-D28K levels.

### 2.1 Inclusion criteria

Articles written in the English language according to the PECOS – Population, Exposure, Comparator, Outcome, and Study design - were included.

• Population (P): Individuals with renal conditions and associated dental history
• Exposure (E): Salivary Calbindin D28k as biomarker's in individuals undergoing renal dialysis associating it with their oral health.
• Comparator (C): Participants with or without renal conditions with varying oral health conditions
• Outcome (O): Effect of Calbindin D28k on oral health.
• Study Design (S): Clinical trials, case–control studies, *in vitro* studies, cross-sectional studies, or cohort studies published in scientific journals.

### 2.2 Exclusion criteria

Case report's, review articles, book chapter's, thesis and guidelines were not taken into consideration. Furthermore, according to the STROBE criteria, checklist was used to analyse the studies which had to be included in the review. 5 criteria were selected has follows: a) Inclusion criteria b) exclusion criteria c) individuals with diagnosed renal condition and association with oral health d) Dental caries and periodontal health described in individuals with diagnosed renal condition e) mechanism of Calbindin D28k explained. Based on the criteria studies which presented only 4 out of 5 criteria were selected as low-risk bias, 3–7 were considered as moderate-risk bias, and which had only 2 were selected as high-risk bias.

## 3 Literature review

### 3.1 An overview of calbindin-D28K

According to Heizmann et al. (25), the term “Calbindin” refers to a collection of calcium-binding proteins that include Calbindin, Calretinin, and S100G. These proteins are divided into several subfamilies according to differences in the quantity of calcium binding EF hands. The ubiquitous protein Calbindin-D28K, which has a molecular weight of 28 kDa (26–30) was first isolated from the intestinal mucosa of a chick. It is widely distributed in a variety of species, including cows, pigs, horses, guinea pigs, and chicks (31). It has also been reported to be expressed in a variety of tissues, including the kidney, brain, pancreas, bone, GI tract, and endocrine glands, including the pituitary, thyroid, and adrenal glands (32, 33). 0.1%–1.5% of all soluble proteins are found in the brain (34).

Furthermore, it acts as a marker for particular brain neuronal subtypes, particularly those related to motor control and coordination (35). It is located in the cytoplasm of neurons and is only found in the kidney's distal tubule and proximal section of the collecting ducts. It is crucial for preserving calcium homeostasis (70). According to Gross et al. (36), the gene CALB1 encodes the protein Calbindin 1, which is a member of the calcium-binding protein family along with troponin C and Calmodulin. Formerly thought to be a 27 kDa protein, it is now shown to be a 28 kDa protein (27, 37–41). Calbindin is the name given to a class of proteins that bind calcium, which includes Calbindin, Calretinin, and S100G. The EF-hand domain, a common calcium ion binding motif seen in this class of proteins, is essential to the proteins' ability to bind calcium (42). There are two types of calcium binding domains in calbindin: modified domains (2) that have lost their calcium binding activity and active domains (4). It performs the roles of a calcium sensor and buffer. Within the EF-hands found in loops EF1 to EF5, it has the ability to bind four calcium ions (34).

Calbindin-D28K global fold unites to form a single, globular domain. Certain subdomains, such as the EF hands that make up the EF pairings EF (1, 2), EF (3, 4), and EF (5, 6) share characteristics with other EF domains. The creation of the EF-hand loop in EF hands is facilitated by the interaction between the first and second helices of alpha 1 and alpha 2, respectively, inside each EF hand. Interestingly, within each EF-hand, there exist connections between the first helix (a1) and the second helix (a2). These connections help construct an EF-hand loop (the terminology for EF-hands is described in the Methods section).For example, certain residues on a1 of EF1 (Ile19, Trp20, and Phe23) interact hydrophobically with residues on a2 of EF1 (Leu36, Leu39, and Ala46).Asp24 of a1 and Glu35 of a2 make interactions that further facilitate the development of the EF-hand loop. The remaining EF-hands exhibit comparable interactions as well (43). These minute structural features draw attention to how sophisticated Calbindin-D28K s role is in regulating and binding calcium within cells. Calbindin-D28K interacts with other cellular proteins in ways that are interesting to watch. It specifically experiences a major conformational change upon Ca2 + loading, which results in the production of a calcium sensor protein used in cellular functions.

### 3.2 Function of calbindin-D28K

#### 3.2.1 CBD-28 K function in neurons

In reality, Calbindin-D28K is crucial for preserving the calcium homeostasis in neurons, which prevents neuronal degeneration. Its expression levels may fluctuate in various clinical conditions, which may help identify certain diseases when used as a diagnostic marker. For instance, decreased levels of Calbindin-D28K have been connected to neuronal dysfunction and calcium dysregulation in Alzheimer's disease (35). This highlights the significance of Calbindin-D28K as a potential biomarker and therapeutic target for neurodegenerative diseases. Conditions that are pathological emphasize how vital it is to maintain the integrity and functionality of neurons.

#### 3.2.2 Calbindin-D28K function in both the physiological and pathological processes of the renal system

The vitamin D-dependent protein Calbindin-D28K is mostly expressed in the major cells of the collecting and distal convoluted tubules of the kidney, where it plays a crucial role in regulating calcium reabsorption. Any damage to the distal nephron segment can alter the expression of Calbindin and its levels in the urine. Research has verified that urine levels of KIM-1 and Calbindin proteins increase by 11.6 and 2.5 times, respectively, within two days following subsequent cisplatin therapy. Following cisplatin treatment, mice's blood urea nitrogen and serum creatinine levels rise by day three, indicating the progression of acute renal injury.

While intrarenal Calbindin-D28K levels show time-dependent declines on days 3 and 4, there is also a considerable rise in KIM-1 messenger RNAs (mRNAs) and CALB in urine (44). It's interesting to note that early decreases in renal Calbindin levels result in the expression of compensatory mRNA at later times. Overall, the presence of calbindin in urine makes it the best biomarker to determine the prognosis of kidney damage. Calbindin's potential as a urine biomarker for renal illness is supported by its selective release from injured distal tubular cells, where it is involved in calcium reabsorption. Overall, as calbindin is only released from the damaged kidney's distal tubular cells, where it is produced, its presence in urine makes it the ideal biomarker to assess the prognosis of kidney damage (45, 46).

Furthermore, damage can occur to the distal convoluted tubular section of the nephron, and Calbindin can be used to monitor the beginning and course of this injury (45). Dysregulation of calcium and phosphate metabolism due to reduced kidney function in chronic kidney disease, which is also responsible for secondary hyperparathyroidism and vascular calcification. Alteration in Calbindin-D28K expression in chronic kidney disease impacts calcium processing and exacerbates mineral imbalance even more problematic for people undergoing the procedure (47)

#### 3.2.3 Renal dialysis patient's oral health

Common oral health issue: In addition to other oral health problems, patients receiving renal dialysis frequently struggle with xerostomia, gingival inflammation, periodontal disease, and heightened susceptibility to mouth infections. A multitude of factors, including reduced salivary flow, altered salivary pH, and the presence of uremic toxins, may impact these circumstances (3, 48). The general term for microbially-induced inflammation of the tissues supporting teeth is periodontitis, which can finally result in the loss of the tooth, the alveolar bone, and the periodontal attachment. The immune-inflammatory response of the hosts and the subgingival bacteria preserve the homeostasis of the periodontal tissues (49).

#### 3.2.4 Effect of systemic conditions

Oral health is deteriorated by systemic effects of chronic kidney disease and dialysis, including immunosuppression, long-term inflammation, and mineral imbalance. More precisely, anomalies in the metabolism of phosphate and calcium can affect tooth mineralization and periodontal health, suggesting a potential link with calcium-regulating proteins such Calbindin-D28K. The stringency of periodontitis can be impacted by a variety of pathogenic circumstances, which may then have an effect on systemic health. Additionally, periodontitis has been reported to be independently associated with several chronic non-communicable diseases, with less evidence regarding chronic kidney disease (3).

#### 3.2.5 Association of calbindin-D28K with oral health

##### 3.2.5.1 Prospective mechanisms

Calbindin-D28K functions in maintaining calcium homeostasis and may have several effects on dental health. Functioning of Salivary gland: Saliva is produced and secreted by the salivary glands located in the mouth. Saliva moistens food, aids in digestion, and protects teeth from decay by neutralizing acids. It also contains enzymes that begin food breakdown in the mouth before it reaches the stomach for further digestion. By clearing the mouth of food particles and bacteria, saliva also contributes to the health of teeth. Gum disease and cavities are two dental issues that are brought on by low saliva. The way that calcium is handled by salivary glands may be affected by Calbindin-D28K. Saliva's composition and flow are important for maintaining dental health, and this could have an impact on them (50).

Periodontal health: Calbindin-D28K may have an effect on periodontal health via controlling calcium levels. Bone density and periodontal structural integrity may be impacted. Although there is a lot of evidence connecting chronic kidney disease and Periodontitis, some studies also reveal a direction of relationship between the two conditions (51). Common pathogenic pathways linking periodontitis and chronic kidney disease include endothelial dysfunction, oxidative stress, and prolonged, inefficient inflammation. Moreover, immunosuppressive medication, comorbidities, and homeostatic changes in chronic kidney disease may encourage periodontitis. Further randomized controlled trials and prospective studies are needed to fully comprehend the relationship between chronic kidney disease and periodontitis. Clarifying how periodontitis therapy affects renal outcomes should be the main goal of these investigations (3, 52)

Mineralization of tooth: Ameloblasts, odontoblasts, and cementoblasts are highly differentiated cells that generate the highly mineralized tissues that make up mammalian teeth. One important function of Ca2 + deposition in these cells is to facilitate their formation. Ameloblasts have been demonstrated to contain Calbindin-D28K in a number of studies (53–55). Patients on dialysis may have variations in Calbindin-D28K expression, which could affect the teeth's mineralization process and increase their susceptibility to dental caries and other mineralization issues.

### 3.3 Biochemical pathways involving calbindin-D28K

Calbindin-D28K binds intracellular calcium in the cytoplasm and moves it through the cytosol in the direction of the basolateral membrane. The plasma membrane Ca2+-ATPase and sodium-calcium exchanger-1 mediate basolateral calcium extrusion (56). Parathyroid hormone, 1,25(OH)2 D3, calcium consumption, and estrogens all control this active transcellular transport. Calbindin-D28K, Calbindin-D9K, sodium-calcium exchanger, plasma membrane calcium pump, and epithelial calcium channel are among the calcium transport proteins that localize to the distal nephron and are activated by 1,25(OH)2D via the activation of the vitamin D receptor (57). Parathyroid hormone increases Calbindin-D28K and other protein expression to promote renal calcium reabsorption (58).

Calbindin-D28K interacts to Ca2 + and is regulated by 1,25(OH)2D3. Vitamin D regulates Calbindin-D28K in a specific manner. In the kidney and gut, 1,25(OH)2D3 is required for CaBP-28K function.Together, these processes support the ongoing maintenance of strong teeth, healthy gums, and good dental care. Calbindin-D28K is able to regulate intracellular calcium levels, which impact the actions of various inflammatory mediators, thereby modulating inflammatory responses. Calbindin-D28K can aid in preserving healthy gums and averting periodontal disorders by lowering inflammation. The regulation of calcium-dependent activities in salivary glands is influenced by Calbindin-D28K, which in turn affects the secretion and production of saliva. Through the regulation of intracellular calcium levels, which are involved in apoptosis, Calbindin-D28K can affect pathways involved in cell survival. The periodontal ligament and gingiva are two examples of oral tissues that Calbindin-D28K supports in preserving their integrity and general health.

A calcium-binding protein called Calbindin-D28K modifies intracellular calcium levels to control many cell signaling pathways (59). In a number of signaling pathways, calcium ions (Ca2+) play a crucial secondary messenger role. Calbindin-D28K regulates some of the key cell signaling pathways such as by maintaining intracellular Ca2 + levels, activation of calcium-sensitive kinases and phosphatases that modulate the MAPK/ERK pathways (40). The survival of the oral tissues as well as activities like cell proliferation and differentiation are impacted by this control. Furthermore, this protein buffers Ca2+, which stops excessive cytochrome c release and apoptosis, helping to maintain the integrity of the mitochondrial membrane. It controls the amount of intracellular Ca which in turn affects the function of calcium-dependent apoptotic signaling enzymes such as calpains. Calbindin-D28K controls calcineurin activation and, consequently, NFAT nuclear translocation via buffering Ca2 + . This control affects the generation of cytokines, immune cell differentiation, and activation. Calbindin-D28K, in general, affects calcium-dependent processes by adjusting intracellular calcium levels, which in turn regulates cell signaling pathways. For the exact regulation of many physiological processes, such as gene expression, apoptosis, cell proliferation, and immunological responses, it plays a critical role in buffering and transporting calcium.

### 3.4 Clinical evidence

A small number of researchers have specifically looked at the connection between dialysis patients' dental health and Calbindin-D28K. However, there is indirect evidence to support this association from the current research on calcium metabolism in chronic kidney disease and its implications for dental health. Research on salivary calcium levels, periodontal health, and dental caries in dialysis patients may provide important new information about the function of Calbindin-D28K (9).

Mohamed et al. (60) meta analysis found a decrease in intestinal calcium absorption, hence there was an imbalance of calcium in chronic kidney disease and end stage renal disease. The blood's 1,25(OH)2D levels are noticeably below average. Individuals requires therapeutic levels of precursors like vitamin D or intermediary metabolites like 25(OH)D because of their critical role in preserving calcium balance in chronic kidney disease and end stage renal disease subjects. In a chronic kidney disease biochemical study, the study groups exhibited significantly greater levels of altered salivary calcium (Ca), phosphorous (P), urea, sodium (Na), and potassium (K) compared to the control groups. The presence of urea in saliva, as observed in these patients, may be reflected in the elevated levels in dialysis patients connected with renal disease severity and, consequently, salivary flow rate, dental caries prevalence, and calculus deposition (61). A correlation appears to exist between the severity of periodontitis and chronic kidney disease, periodontitis is more severe in chronic kidney disease patients than in the general population (62), as periodontitis gets severe, there is increase in chronic kidney disease severity (63), and the duration of renal replacement therapy is linked to periodontitis severity in patients receiving peritoneal dialysis (64).

### 3.5 Calbindin-D28K in renal dialysis

Monitoring Calbindin-D28K levels in renal dialysis patients can help healthcare providers assess their calcium status and make appropriate adjustments to their treatment plan. Understanding the role of Calbindin-D28K in calcium regulation is crucial for optimizing bone health and overall well-being in this patient population (27).

Additionally, Hunag et al. 2019 study has been demonstrated that the kidneys of streptozotocin-induced diabetes rats (65) and OVE26 diabetic mice (66) have dramatically altered Calbindin-D28K expression. Another study demonstrated that Calbindin-D28K buffering effects on the control of intracellular calcium concentrations protected against cyclosporine A-induced renal proximal tubular cell cytotoxicity (67). Additionally, Rabinovitch et al., 2001 discovered that Calbindin-D28K inhibits the production of free radicals, hence providing protection against cytokine-mediated β-cell death (68). These results showed that Calbindin-D28K may contribute to the amelioration of diseases in a number of target organs, including the kidney (40).

**Figure 1:**
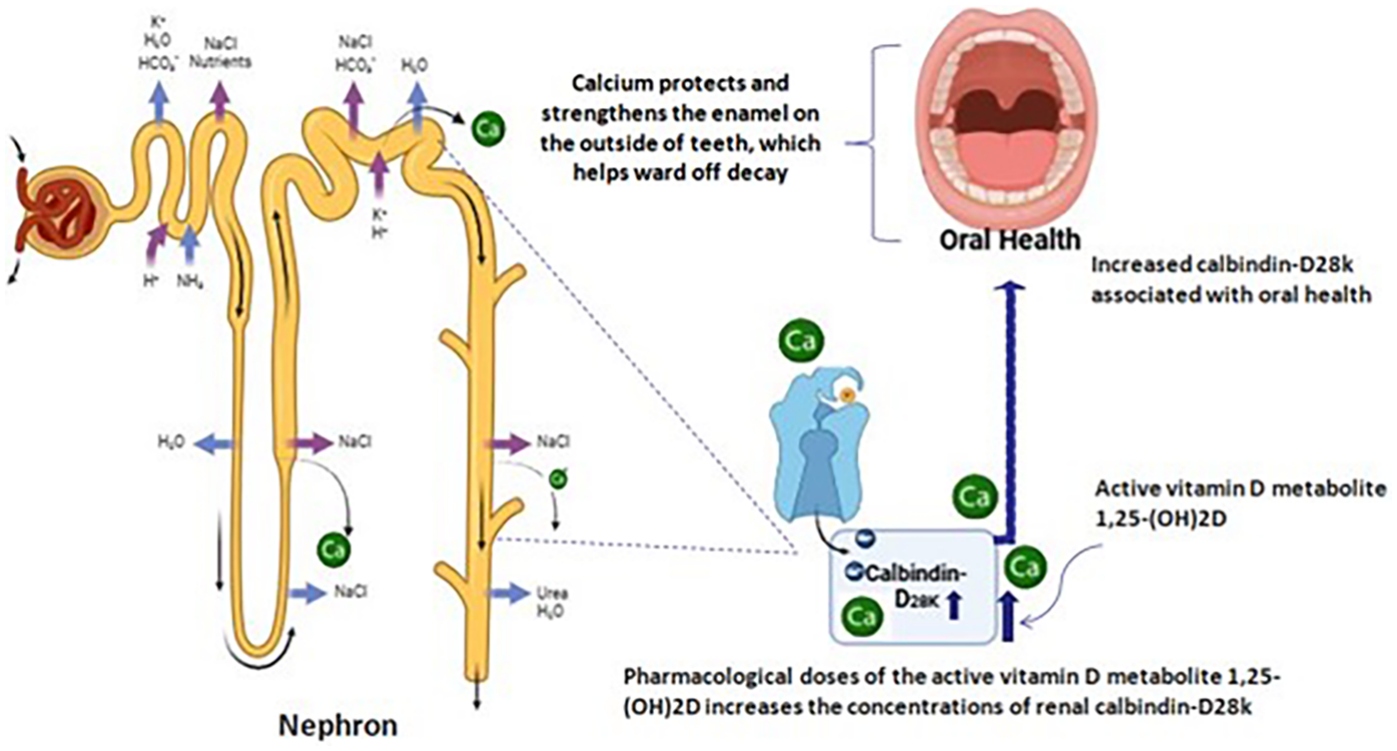
Relationship between oral health and renal Calbindin-D28K in renal dialysis: Calbindin-D28K is known to be present inhigh concentration in distal convoluted tubules, connecting tubules and collecting ducts of the nephron, where it is involved in the absorption of calcium.

Figure 1 Relationship between oral health and renal Calbindin-D28K in renal dialysis: Calbindin-D28K is known to be present in high concentration in distal convoluted tubules, connecting tubules and collecting ducts of the nephron, where it is involved in the absorption of calcium. Pharmacological doses of the active vitamin D metabolite 125-(OH) 2D increases the concentration of renal Calbindin-D28K. Calcium ion is part of the teeth mineral content and interferes in the des-mineralization processes. When oral fluids are supersaturated with this ion, decayed is unfavored. Hence, the availability of the calcium required for the healthy teeth will be limited. This disturbs the oral health.

Future Directions: Because the time of renal failure discovery and death is closely associated, early identification and fast treatment of pharmacological features that can identify Calbindin-D28K's in treatment of oral manifestation in chronic kidney disease. To completely comprehend the mechanisms underlying this link and investigate potential therapies to enhance oral health outcomes in people receiving renal dialysis, more study is required. Rehab patients may experience improved general health and quality of life if these problems are resolved. It may be possible for healthcare professionals to enhance the general health and quality of life of people receiving renal dialysis by attending to oral health concerns. Calbindin-D28K's detrimental effects on this population's oral health may be lessened by putting preventive measures into place and receiving routine dental treatment.

Research Gaps: There are still many unanswered questions regarding the function of Calbindin-D28K in dialysis patients' dental health. Future studies ought to concentrate on: Mechanistic Studies: Examining the chemical pathways by which Calbindin-D28K affects dental health. Clinical Research: Observational and interventional studies are being carried out to investigate the connection between oral health outcomes and Calbindin-D28K expression in dialysis patients. Biomarker Development: Assessing Calbindin-D28K's potential as a biomarker to anticipate and track oral health problems in individuals with chronic kidney disease and dialysis.

Final considerations: In addition to being vital to calcium homeostasis, Calbindin-D28K is also deeply ingrained in the pathophysiology of chronic kidney disease and difficulties associated with dialysis. Substantial systemic consequences result from these changed levels, and oral health may be affected. Individuals receiving renal dialysis frequently show abnormalities in their metabolism of calcium, which is connected to periodontal and dental problems such tooth decay and gum disease. This link implies that a higher occurrence of certain dental disorders may be caused by lower levels of Calbindin-D28K in these patients. Increased Calbindin-D28K levels in dialysis patients have been linked to modifications to the composition of their saliva. Such alterations may make dental issues worse in this population because saliva is essential for preserving oral health. Enhanced clinical procedures may result from a better understanding of the relationship between Calbindin-D28K levels and oral hygiene. Developing comprehensive treatment programs that address systemic and oral health requires a close connection between nephrologists and dentists. The negative impacts dialysis patients have on their oral health may be lessened with regular dental checkups, preventative measures, and customized oral hygiene habits. More thorough research is required, even though initial findings point to a connection between renal dialysis patients' dental health and their Calbindin-D28K levels. Subsequent research endeavors ought to clarify the exact pathways by which modified quantities of Calbindin-D28K impact oral health and devise evidence-based guidelines for the comprehensive management of patients undergoing dialysis. The information points to important consequences for oral health resulting from the modification of Calbindin-D28K levels during renal dialysis. For those receiving renal dialysis, addressing these consequences through focused research and interdisciplinary collaboration can improve overall health and quality of life (69).
